# Data-Driven Modeling for Species-Level Taxonomic Assignment From 16S rRNA: Application to Human Microbiomes

**DOI:** 10.3389/fmicb.2020.570825

**Published:** 2020-11-12

**Authors:** Ho-Jin Gwak, Mina Rho

**Affiliations:** ^1^Department of Computer Science and Engineering, Hanyang University, Seoul, South Korea; ^2^Department of Biomedical Informatics, Hanyang University, Seoul, South Korea

**Keywords:** 16S rRNA, microbial community, differential composition, operational taxonomic units, taxonomy assignment

## Abstract

With the emergence of next-generation sequencing (NGS) technology, there have been a large number of metagenomic studies that estimated the bacterial composition via 16S ribosomal RNA (16S rRNA) amplicon sequencing. In particular, subsets of the hypervariable regions in 16S rRNA, such as V1–V2 and V3–V4, are targeted using high-throughput sequencing. The sequences from different taxa are assigned to a specific taxon based on the sequence homology. Since such sequences are highly homologous or identical between species in the same genus, it is challenging to determine the exact species using 16S rRNA sequences only. Therefore, in this study, *homologous species groups* were defined to obtain maximum resolution related with species using 16S rRNA. For the taxonomic assignment using 16S rRNA, three major 16S rRNA databases are independently used since the lineage of certain bacteria is not consistent among these databases. On the basis of the NCBI taxonomy classification, we re-annotated inconsistent lineage information in three major 16S rRNA databases. For each species, we constructed a consensus sequence model for each hypervariable region and determined *homologous species groups* that consist of indistinguishable species in terms of sequence homology. Using a *k*-nearest neighbor method and the species consensus sequence models, the species-level taxonomy was determined. If the species determined is a member of *homologous species groups*, the species group is assigned instead of a specific species. Notably, the results of the evaluation on our method using simulated and mock datasets showed a high correlation with the real bacterial composition. Furthermore, in the analysis of real microbiome samples, such as salivary and gut microbiome samples, our method successfully performed species-level profiling and identified differences in the bacterial composition between different phenotypic groups.

## Introduction

Metagenomics has been widely used to analyze microbial communities without cultivating strains (Breitbart et al., 2003; Schloss and Handelsman, 2003; Handelsman, 2004; Petrosino et al., 2009; Qin et al., 2010; Peng et al., 2019; Yang L. et al., 2019; Brumfield et al., 2020; Chung et al., 2020; Khachatryan et al., 2020). Moreover, the 16S ribosomal RNA (16S rRNA) gene has been regarded as an informative resource for the identification of the species and the estimation of bacterial composition as it has both well-conserved and hypervariable regions among different species. Thus, the conserved regions can be used as primers to target specific hypervariable regions using targeted amplicon sequencing (Petrosino et al., 2009), whereas the hypervariable regions can be used to identify bacterial taxonomy using the sequence similarities between different species. Although the 16S rRNA gene is a useful material to identify bacteria, it is challenging to completely discriminate species since 16S rRNA genes are identical or highly homologous between some different species. Genome comparisons by DNA–DNA hybridization or genome sequence comparison (ANI analyses) were needed to assign an exact species (Cho and Tiedje, 2001; Ciufo et al., 2018).

Using 454 pyrosequencing (Petrosino et al., 2009; Cummings et al., 2013) and Illumina MiSeq technology (Wen et al., 2017; Ravi et al., 2018; Sessou et al., 2019), 16S rRNA analysis pipelines were built to estimate the bacterial composition of different species (Turnbaugh et al., 2007; Jumpstart Consortium Human Microbiome Project Data Generation Working Group, 2012). While attempts are being made to analyze the entire 16S rRNA sequence via long-read sequencing using PacBio (Quail et al., 2012) or Oxford Nanopore (Winand et al., 2019) technology, the high error rates and costs limit their practical utility. When estimating bacterial composition using targeted amplicon sequencing, the results might differ depending on the choice of hypervariable regions, such as V1–V2 or V3–V4. Therefore, selecting appropriate hypervariable regions for analysis is important. Several studies have been conducted to investigate the manner in which the analysis of different variable regions affects the estimation of bacterial composition (Sun et al., 2013; Johnson et al., 2019).

The 16S rRNA analysis pipeline involves preprocessing, clustering [operational taxonomic units (OTU) picking], assigning taxonomy, and estimating the bacterial composition. Although most of the sequencing errors are filtered out at the preprocessing step, there are still some sequencing errors that remain. To overcome these errors and strain variations, processed reads are clustered into OTUs using a 97 or 99% sequence similarity threshold. Since sequences belonging to the same OTU are considered to be derived from the same clade, OTU clustering directly affects the estimation of bacterial composition. Therefore, several clustering algorithms have been developed to overcome strain variation and sequencing errors. For example, the UPARSE algorithm (Edgar, 2013) clusters sequences on the basis of sequence similarity, whereas the Minimum Entropy Decomposition (MED) (Eren et al., 2015) and DADA2 (Callahan et al., 2016) algorithms cluster sequences via the association of position-specific variations. For taxonomy assignment, classifiers such as MEGAN (Huson et al., 2007), RDP naïve Bayesian classifier (Wang et al., 2007), Kraken (Wood and Salzberg, 2014), and SPINGO (Allard et al., 2015) were developed. Thus, not only the classifier but also the 16S rRNA database is important for accurate taxonomical classification. There are currently three major 16S rRNA databases that are widely used, namely GreenGenes (DeSantis et al., 2006), SILVA (Quast et al., 2013), and RDP (Cole et al., 2014). However, although new bacterial taxa continue to be reported, these three databases have not been updated for over 2 years. Furthermore, the lineage of some bacteria is not consistent among these three databases (Balvociute and Huson, 2017; Edgar, 2018a).

In this study, we re-annotated the inconsistent or mislabeled taxa in the three 16S rRNA databases on the basis of the NCBI taxonomy classification. The 16S rRNA sequences were combined from the re-annotated GreenGenes, SILVA, and NCBI databases to include species that exist exclusively in each database or were recently annotated. In the evaluation of taxonomy classification, the classifier trained with all three databases showed the best accuracy in terms of precision and recall rates. Moreover, *taxonomic separability* was measured for the V1–V2 and V3–V4 hypervariable regions at the genus and species level. For each species, we constructed consensus sequences for each hypervariable region and determined indistinguishable species. By comparing the consensus sequences of each species, *homologous species groups* in which the species share high similarity were constructed for each hypervariable region, which was then used for the species-level taxonomy assignment. The evaluation performed using simulated datasets and mock datasets showed a high correlation with the real bacterial composition. Moreover, when analyzing real microbiomes, such as the salivary and gut microbiome, our method successfully performed species-level profiling to identify differences in bacterial composition between different phenotypic groups.

## Materials and Methods

### Re-annotating the 16S rRNA Sequence Databases

To investigate the taxonomy consistency, GreenGenes v13.5, SILVA v132, and RDP v11.5 databases were used. As quality control, sequences whose length range in three times the standard deviation from the mean without any ambiguous nucleotide (e.g., N) were used. Out of 1,242,330, 1,861,373, and 3,196,041 sequences obtained from GreenGenes, SILVA, and RDP databases, respectively, 1,191,315, 1,779,305 and 1,559,121 sequences were retained for the re-annotation after quality control process (Supplementary Table 1).

To apply the latest version of NCBI taxonomy, NCBITaxa class in the ete3 python package (Federhen, 2012; Huerta-Cepas et al., 2016) was used with NCBI taxdump downloaded on January 3, 2020. The taxonomy tree with seven taxonomic ranks (superkingdom, phylum, class, order, family, genus, and species) was used in this re-annotation.

**FIGURE 1:**
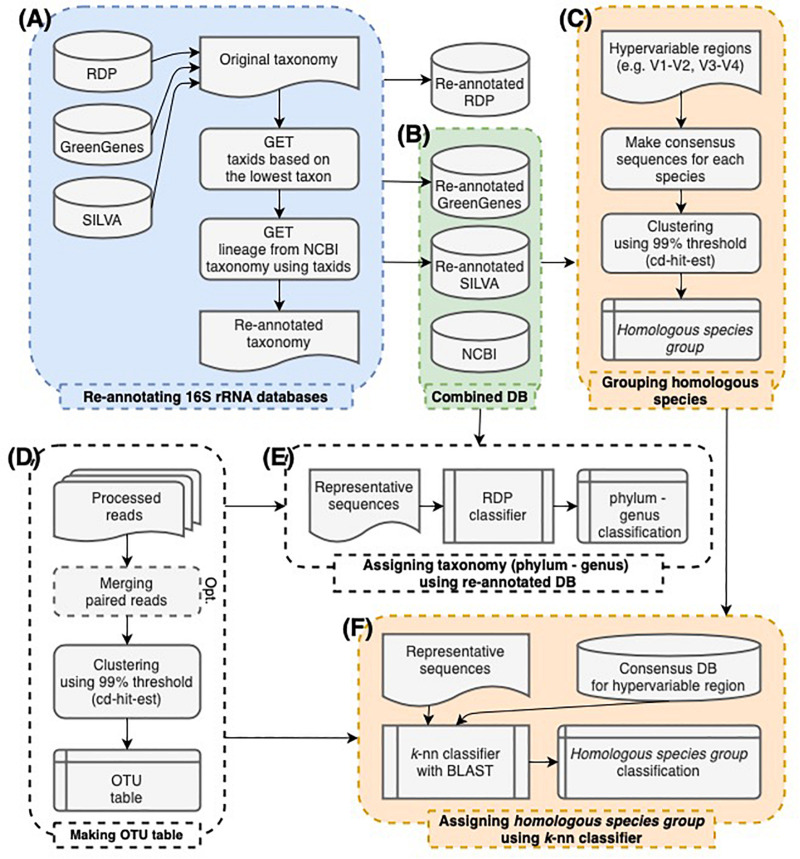
The workflow of this study. **(A)** The taxonomy of 16S rRNA sequences in GreenGenes, SILVA, and RDP databases was re-annotated according to the NCBI taxonomy. **(B)** GreenGenes, SILVA, and NCBI databases were combined. RDP database was excluded since they have no species annotation. **(C)** Consensus sequences of each species were made by hypervariable region sequences extracted from the combined databases. By clustering consensus sequences within 99% sequence similarity, *homologous species groups* were established. **(D)** OTU tables were made in a conventional manner. **(E)** The taxonomy assignment from phylum to genus-level was processed by the RDP naïve Bayesian classifier re-trained using the combined database. **(F)** The species-level classification was processed by searching sequences against the consensus database. Sequences were labeled as the representative species of the *homologous species group* that includes the best hit of the sequence.

Each 16S rRNA sequence was re-annotated using the taxon at the lowest taxonomy rank in the database (Figure 1 and Supplementary Figure 1). To identify the lowest rank, the provided taxa were searched from species to superkingdom. For each rank, the taxid was returned if it was found using the get_name_translator() function in NCBITaxa class. Otherwise, that rank was skipped. When the species name was specified with the strain name at the species rank, only the species name was used. Since *Escherichia* and *Shigella* species have essentially identical 16S rRNA sequences, sequences labeled as *Escherichia* or *Shigella* were collectively labeled as *Escherichia.Shigella*.

### Gathering the 16S rRNA Sequences From the Genomes in the NCBI RefSeq Database

The genomes assembled at the complete-level or chromosome-level were downloaded from the ftp site of the NCBI RefSeq database^1^. The information for each genome is listed in the *assembly_summary_refseq.txt* file (downloaded on July 16, 2019). In the generic feature file (GFF), the regions where the feature is described as “rRNA” and the product as “16S ribosomal RNA” were identified as the 16S rRNA sequences and extracted from the genome. Thus, we obtained 78,270 16S rRNA sequences from the 16,337 genomes analyzed. As quality control, the same filtering step was performed for the sequences extracted and 77,803 16S rRNA sequences were retained to train the classifier and generate consensus models.

### Simulating the Hypervariable Regions From the 16S rRNA Sequences

The 27F/308R and 337F/806R primer pairs are widely used to target the hypervariable regions V1–V2 and V3–V4, respectively, for Illumina MiSeq amplicon sequencing. The fragment sequences of the hypervariable regions were simulated by extracting the sequences between the forward and backward primers from the 16S rRNA sequences using cutadapt (Martin, 2011). Moreover, an error level of 20% (i.e., 2–3 nt mismatches) was allowed when matching the primer sequences. The mean and standard deviation of the extracted fragment length were also calculated. Fragments longer or shorter than twice the standard deviation from the mean value were ignored. Fragments containing “N” were also ignored (Supplementary Table 2).

### Constructing *Homologous Species Groups* for Each Hypervariable Region

To determine which species are distinguishable by their 16S rRNA sequences, sequence similarities between species belonging to the same genus were calculated. A consensus sequence of the strains belonging to the same species was obtained using the “cons” function in EMBOSS v6.6.0 (Olson, 2002) with the default parameter settings. Pairwise sequence similarities were measured between the consensus sequences of each pair of species using the Needleman–Wunsch algorithm (Needleman and Wunsch, 1970) implemented in the “needle” function in EMBOSS v6.6.0 with the default parameter settings.

On the basis of the sequence similarity of the consensus sequences, *homologous species groups* that shared 99% or higher sequence similarity were constructed. The species in a *homologous species group* were considered indistinguishable by their 16S rRNA sequences. To name the *homologous species group*, the species in the group with the largest number of strains were selected and extended with a “+” sign.

### Simulating Amplicon Sequences From the Bacterial Genomes

Amplicon sequences for the V3–V4 hypervariable region were simulated from the bacterial reference genomes using Grinder (Angly et al., 2012). To target the V3–V4 hypervariable region, the 337F (CCTACGGGAGGCWGCAG) and 806R (GACTACHVGGGTMTCTAAT) primer sequences were used. For abundance models, the uniform, linear, and power-law with parameter 1 and 2 models were used. Amplicon sequences were simulated with a uniform 0.5% error model (-md uniform 0.5) and a length distribution of 421 ± 11 bp (-rd 421 uniform 11). Only the forward strands were used (-un 1), and the coverage fold was set to 1,000 (-cf 1000). Moreover, we considered copy number bias but not genome length bias (-cb 1 and -lb 0). All other parameters (i.e., those not mentioned above) were set as default.

### Preprocessing of the Illumina Amplicon Sequencing Reads

The 16S rRNA genes were sequenced using the Illumina MiSeq sequencer, and paired-end reads were generated and merged on the basis of their overlapping region. Each read pair was assembled using FLASH (Magoc and Salzberg, 2011) with the default parameter settings except for a minimum overlap of 20 bp (-m 20) and maximum overlap of 300 bp (-M 300). Assembled contigs (including “N”) were removed using an in-house script. Merged fragments longer than twice the standard deviation from the mean of the hypervariable region length (mean and standard deviation of the V3–V4 region were 421 and 11 nt, respectively) were also removed using Sickle. The mean and standard deviation of the V3–V4 region length were calculated from the sequences in the GreenGenes database.

### Constructing the OTUs and Determining Their Taxonomy Assignment

The classification of the 16S rRNA sequence was performed according to the conventional classification approach (Figure 1). Preprocessed reads were clustered into OTUs using cd-hit-est (Fu et al., 2012). Cd-hit-est was used with the following parameter settings: no memory limitation (-M 0), word size 10 (-n 10), cluster into the most similar cluster (-g 1), and a 99% sequence similarity threshold (-c 0.99). The other parameters were set as default. Each representative sequence was classified using the RDP naïve Bayesian classifier trained with our combined database.

## Results and Discussion

### Refinement of Inconsistent Taxonomy Annotation in 16S rRNA Databases

Using the 16S rRNA sequences from three major 16S rRNA databases, we investigated the consistency of the taxonomic lineage annotation. When we compared the taxonomic lineage annotations provided by the three 16S rRNA databases, we found that the same genus or species was often annotated with a different lineage. Out of the 1,122, 4,985, and 2,191 genera included in the GreenGenes v.13.5, SILVA v.132, and RDP v.11.5 databases filtered, respectively, 183, 2,794, and 68 were exclusive to each database (Supplementary Figure 2A). Notably, out of the 853 genera included in all three databases, only 288 were annotated with the same lineage. Moreover, 112 genera were annotated with different lineages in all three databases. For example, the order of *Mycobacterium* was annotated as Actinomycetales in the GreenGenes and RDP databases but as Corynebacteriales in the SILVA database. The order of *Corynebacterium* was also annotated as Actinomycetales in the GreenGenes and RDP database but as Corynebacteriales in the NCBI taxonomy classification. Taxonomy reclassification also resulted in inconsistent taxonomic lineage annotation among the three databases. For example, *Propionibacterium* was originally identified as *Bacillus* but was later renamed as *Propionibacterium* (Douglas and Gunter, 1946). However, it was recently reclassified as *Cutibacterium* (Dreno et al., 2018).

We re-annotated the three major 16S rRNA databases based on NCBI taxonomy since inconsistencies between the databases could produce different bacterial composition profiles depending on the choice of database. Using the sequences filtered from GreenGenes, SILVA, and RDP databases, 667,528 (56%), 907,944 (51%), and 1,275,668 (82%) sequences were re-annotated in this study, respectively (Supplementary Table 1). As a result, we obtained 879 genera with the same lineage annotation among three databases (Table 1, Supplementary Figure 2B), compared to the 288 genera identified before the refinement step (Supplementary Figure 2A). Only four genera existed exclusively in the GreenGenes, 15 genera in the RDP, and 955 genera in the SILVA database (Supplementary Figure 2B).

**TABLE 1:** The number of taxa for each taxonomic rank after re-annotation.

	Green genes	SILVA	RDP	NCBI
Superkingdom	1	1	1	1
Phylum	40	62	49	40
Class	72	85	73	72
Order	163	210	173	164
Family	355	495	380	358
Genus	1,030	3,239	2,154	1,206
Species	570	15,335	0	3,029
Number of sequences	1,191,315	1,779,305	1,559,121	77,869

After the re-annotation, the sequences from GreenGenes and SILVA databases were used in our classification method (Figure 1B). The RDP database was excluded since species-level annotation was not provided. In addition, the 16S rRNA sequences extracted from the complete genomes in the NCBI RefSeq database were included. In total, 823,937, 1,306,532, and 77,410 sequences with the genus-level annotation from GreenGenes, SILVA, and NCBI, respectively, were used in our classification method (Supplementary Table 1).

### Genus-Level Profiling Using the Combined Database

**FIGURE 2:**
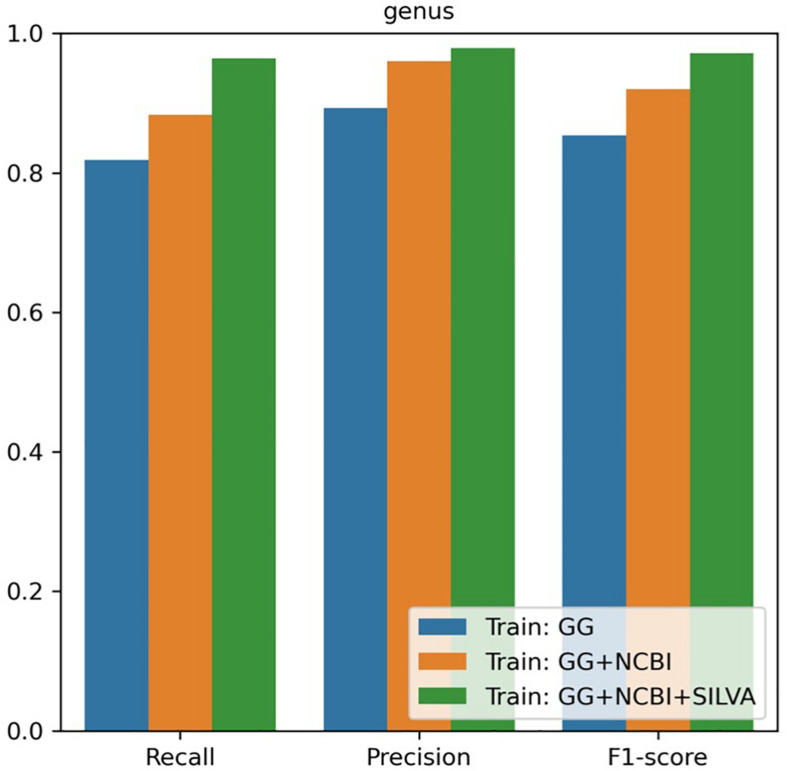
Performance of the genus-level classification with the combined database.

For the genus-level taxonomy assignment, the RDP naïve Bayesian classifier was retrained with the sequences re-annotated in this study. Classifiers were tested using the V3–V4 region sequences extracted from the NCBI database. In the evaluation, the classifier trained with our combined database showed the best performance in terms of precision and recall rates from the phylum to genus level (Figure 2 and Table 2). Notably, the classifier trained with one database (i.e., GreenGenes) had precision and recall rates of 89.33 and 81.85%, respectively, whereas the classifier trained with all three databases had precision and recall rates of 97.88 and 96.39%, respectively.

**TABLE 2:** Accuracy of the taxonomy classification when using the combined database.

	Recall	Precision	F1-score
Superkingdom	1	1	1
Phylum	0.9997	0.9995	0.99962
Class	0.9896	0.9989	0.99422
Order	0.9923	0.9981	0.99517
Family	0.9659	0.9954	0.98045
Genus	0.9832	0.9666	0.97482
Species	0.7696	0.7994	0.78423

To evaluate the classification performance for the newly annotated bacteria, the gut microbiome of mice (Chung et al., 2020) were profiled using the classifier trained with our combined database (Supplementary Figure 4). In the previous report, the profiling of the bacterial composition of the samples using metagenomic reads revealed that Muribaculaceae and its genera were the most abundant taxon, whereas profiling via the 16S rRNA amplicon sequencing reads showed that Barnesiellaceae was the most abundant. This difference was explained by the different versions of the databases used (Chung et al., 2020). Sequences annotated with these two genera were not included in the GreenGenes database and RDP database, since *Muribaculum* and *Duncaniella* were first reported in the NCBI repository in July 2016 and March 2018, respectively. Notably, the classifier trained in our study correctly predicted the sequences as Muribaculaceae at the family rank, suggesting that the relative abundance of *Duncaniella* is similar to that obtained via metagenomic analysis. Although *Duncaniella* was well classified, *Muribaculum* was still reported with a low confidence score. This result suggests that there might be some genera belonging to the Muribaculaceae family that are still unknown.

### Species-Level Profiling Using *Homologous Species Groups*

In conventional microbiota profiling, reads are clustered into OTUs based on sequence similarity. Most OTUs are created using a 97 or 99% sequence similarity threshold. These thresholds are based on the empirical observation of 94% or higher 16S rRNA sequence similarity within a genus and 97% or higher 16S rRNA sequence similarity within a species (Schloss and Handelsman, 2005). Note that, many studies have reported that species cannot be completely discriminated using such thresholds (Stackebrandt, 2006; Edgar, 2018b). We measured the *taxonomic separability* (i.e., how well different taxa are separately assigned to different OTUs) using the V1–V2 region, the V3–V4 region, and the entire 16S rRNA gene. OTUs were created using a 99% sequence similarity threshold to measure the proportion of OTUs that were assigned to multiple taxa (Supplementary Figure 5).

Most of the OTUs created consisted of sequences from one genus, whereas multiple species were assigned to the same OTU. Out of the 84,169, 127,223, and 179,039 OTUs created from the V1–V2 region, the V3–V4 region, and the entire 16S rRNA gene in the GreenGenes database, 3.58, 1.51, and 0.29% of the OTUs contained multiple species, respectively (Supplementary Figure 5A). In the SILVA database, out of the 118,404, 191,585, and 299,556 OTUs created, 20.26, 25.62, and 18.94% contained multiple species, respectively. Moreover, in the 16S rRNA gene sequences obtained from the NCBI database, 13.01, 19.54, and 13.44% of the 3,137, 2,746, and 3,987 OTUs created contained multiple species, respectively. While most of the sequences from different genera were assigned to different OTUs, almost half of the sequences from different species were assigned to the same OTU in the SILVA and the NCBI database (Supplementary Figure 5B). This result indicates that reads from such species are clustered together when OTUs are created using a 99% sequence similarity threshold.

To investigate *species separability* using the 16S rRNA sequences, a species network was constructed with the sequences of the V3–V4 region from our combined database (Supplementary Figure 6). In the network, each node is a consensus sequence of a species. If two species share 99% or higher sequence similarity, the nodes of those species were connected. Notably, many species from the same genus were clustered owing to the fact that their 16S rRNA sequences have 99% or higher similarity. Among the *Staphylococcus* species, seven groups were clustered, the largest of which consisted of 10 species (Supplementary Figure 6A). Moreover, 15 groups were clustered from the *Streptococcus* species, the largest of which consisted of eight species (Supplementary Figure 6B). The *homologous species groups* were constructed from the network analysis, which corresponded to the connected components in the graph.

**FIGURE 3:**
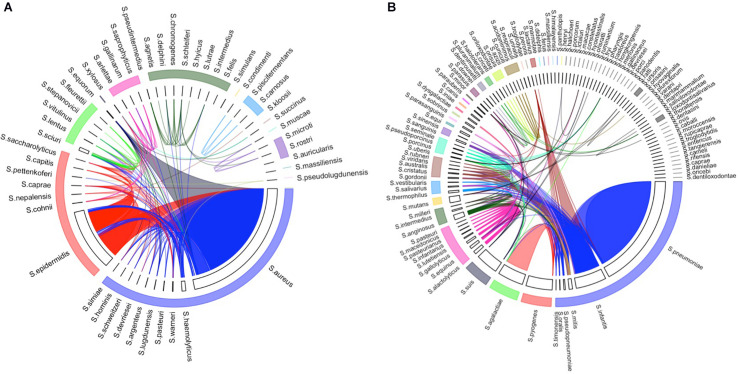
*Homologous species groups* of the species in **(A)** *Staphylococcus* and **(B)** *Streptococcus*. The width of each track is proportionate to the number of non-redundant strains included in the database. *Homologous species groups* (i.e., species whose consensus sequences have more than 99% sequence similarity) were labeled with the same color. Strains that share 99% or higher sequence similarity in different species were linked by an edge.

In the *homologous species groups*, the consensus sequences of the included species had 99% or higher sequence similarity. Figure 3 shows the *homologous species groups* in the arc of the same color, which resulted from the network analysis of two genera, *Staphylococcus* and *Streptococcus* (Supplementary Figure 6). Notably, some strain-level heterogeneity (i.e., 99% or higher sequence similarity between strains in different *homologous species groups*) was also observed (Figure 3). For example, some sequences belonging to *Staphylococcus aureus* and *Staphylococcus epidermidis* (labeled in blue and red) were connected. Such strain-level heterogeneity could be caused by either distinct strains in a specific species or incorrect annotation.

To assign a species-level taxon to the OTUs, the representative sequence of each OTU was searched against our species consensus sequence database using BLAST search (Altschul et al., 1990). Similar to the *k*-nearest neighbor method, the species was determined by considering the most *k* homologous species. In this study, *k* was set to 1 among the sequences with >97% sequence similarity and an e-value of <1.0e-10. When no hit met the criteria, it was reported as unclassified. If the assigned species were from the *homologous species groups*, the query sequence was labeled as the name of the *homologous species group*.

### Evaluation Using Simulated Datasets

To test the performance of our species-level profiling method, simulated datasets were generated using a set of bacteria reported as the constituents of the Human Microbiome Project (HMP) gut microbiome (Supplementary Table 3). The reported strains were downloaded from the NCBI RefSeq database, and the non-existing or updated strains were replaced with the latest strains of the same species. *Candida albicans* ATCC MY-2876 was not included since it is a fungus. Four simulated datasets were generated with the abundance models of uniform, linear, and power-law parameters with 1 and 2 (Supplementary Table 3). *Methanobrevibacter* and *Propionibacterium* (*Cutibacterium*) were excluded from the simulation since the 806R primer could not extract the region sequences from their genomes. The simulated datasets were analyzed using our species-level profiling method.

**FIGURE 4:**
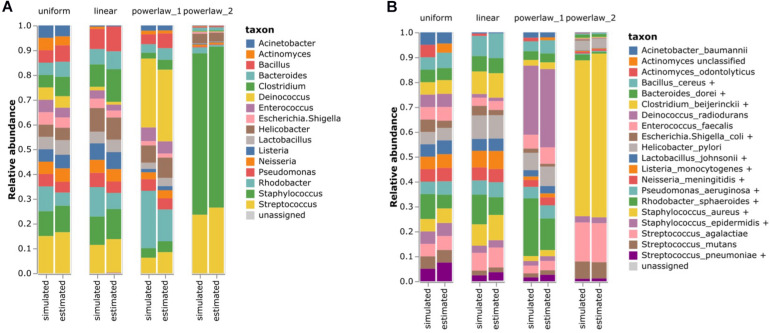
Estimating the bacterial composition of the simulated datasets. **(A)** Genus level and **(B)** species level profiling. Simulated datasets with four different abundance models were analyzed using the proposed 16S rRNA classification pipeline. The names of the species that were contained in the simulated datasets were re-annotated according to the *homologous species groups* of the V3–V4 hypervariable region to compare the results.

Regardless of the abundance model, the genus-level composition was almost perfectly profiled using our method (Figure 4A). Among the species in the simulated dataset, *Bacillus cereus*, *Bacteroides vulgatus, Clostridium beijerinckii*, *Escherichia coli*, *Lactobacillus gasseri*, *Listeria monocytogenes*, *Neisseria meningitidis*, *Pseudomonas aeruginosa*, *S. aureus*, *S. epidermidis*, and *Streptococcus pneumoniae* created the *homologous species groups* with other species. For instance, the V3–V4 region of *B. cereus* was identical to that of *Bacillus mobilis*. These species are technically indistinguishable in terms of their V3–V4 region. Similarly, the V3–V4 sequence of *S. pneumoniae* differs by only one nucleotide from that of *Streptococcus infantis*. Notably, our method based on the *homologous species groups* was able to accurately estimate the species-level composition in the simulated datasets (Figure 4B). Pearson’s correlation coefficient values between the simulated and estimated bacterial composition were 0.9781 and 0.9790 for the genus- and species-level classification results. Therefore, our *homologous species groups* method could reasonably perform accurate species-level profiling.

### Evaluation Using Mock Datasets

**FIGURE 5:**
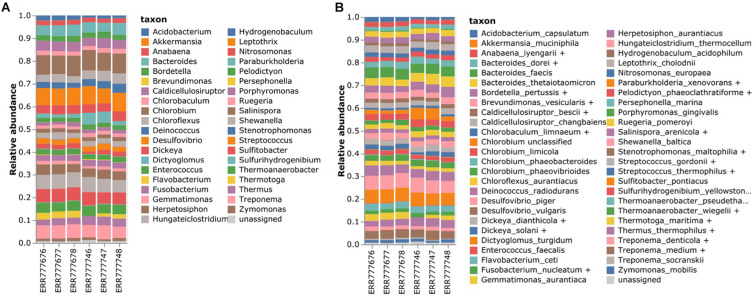
Estimating the bacterial composition of the mock datasets. **(A)** Genus level and **(B)** species level profiling. Six mock samples were analyzed using the proposed 16S rRNA classification pipeline. The names of the species that were contained in the mock datasets were re-annotated according to the *homologous species groups* of the V3–V4 hypervariable region to compare the results.

Six mock datasets consisting of 49 bacteria and 10 archaea (Supplementary Table 4) were downloaded from the EBI sequence repository^2^ (Schirmer et al., 2015). The V3–V4 region was sequenced by Illumina MiSeq2 using the 341F forward primer and two kinds of reverse primers (806rcb and 805RA). The mock datasets were analyzed using our method (Figure 5). Since this mock data set provided a list of microbiome constituents without their relative abundance, we evaluated the results of our method by checking whether the specified genus and species were identified.

In total, 31 out of 38 genera were identified, accounting for an average of 90.7% of the microbiota population. In the case of *Burkholderia*, there were reads classified as *Paraburkholderia*. Moreover, *Anaerocellum* could not be identified owing to the lack of databases. On an average, 5.9% of the reads were misclassified as *Anabaena*, *Brevundimonas*, *Dickeya*, *Flavobacterium*, *Hungateiclostridium*, *Stenotrophomonas*, and *Streptococcus*. For the species-level classification, 31 out of 41 species were identified, of which 21 were assigned with specific species and 10 were assigned with *homologous species groups*. In total, 73.21% of the microbiota population on average was profiled at the species level. However, six species, namely *Anaerocellum thermophilum*, *Burkholderia xenovorans*, *Clostridium thermocellum*, and *Erwinia chrysanthemi*, could not be identified owing to the lack of databases.

### A Case Study Using the Salivary Microbiome

**FIGURE 6:**
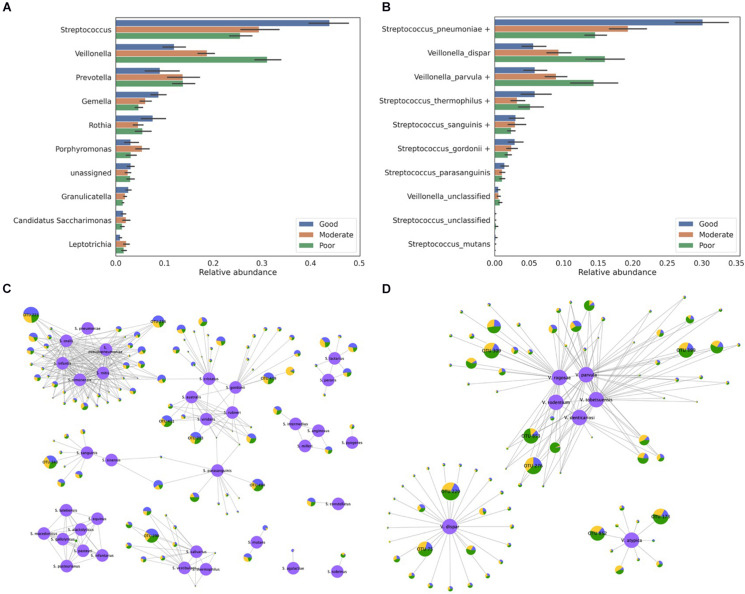
Classification of the salivary microbiome samples obtained from three different hygiene index groups. **(A)** The relative abundance of the top 10 abundant genera and **(B)** the relative abundance of the species belonging to the *Streptococcus* and *Veillonella* genera are presented. The assignment results for **(C)** the *Streptococcus* and **(D)** the *Veillonella* genera are represented by the network. Reference sequences are colored in purple and labeled using their species name. Each node except the reference node represents one OTU, and the bacterial composition of samples from the three hygiene index groups is plotted as a pie-chart (blue for good, orange for moderate, and green for poor). All OTUs are represented as being proportional to their log-scaled size, and all OTUs with a size of 10,000 or more are labeled with their OTU number.

In total, 90 salivary microbiome samples stratified by the oral hygiene index were downloaded from the DDBJ Sequence Read Archive (SRA) under the accession number DRA005425. A previous study reported that *Streptococcus* and *Veillonella* were the most abundant genera in these samples and that their proportions are associated with the hygiene index (Mashima et al., 2017). However, details regarding species-level information were not provided. To profile the species-level composition, we re-analyzed the same salivary microbiome samples (Figures 6A,B). Notably, all of the *Streptococcus* and *Veillonella* OTUs were assigned to a species or *homologous species groups* (Figures 6C,D). With the exception of a few OTUs, most of the OTUs were assigned to the species groups. The *S. pneumoniae* group was identified as the most abundant species among all samples. Moreover, although the *S. pneumoniae* group was identified in both the good and poor hygiene groups, its abundance in the good hygiene group was more than twice that of the poor hygiene group.

In total, eight major *Streptococcus* OTUs were identified from the sample data by considering the size of the OTUs (number of reads in OTU >10,000): two OTUs with the *S. pneumoniae* group, three OTUs with the *Streptococcus gordonii* group, one OTU with the *Streptococcus sanguinis* group, one OTU with the *Streptococcus thermophilus* group, and one OTU with the *Streptococcus parasanguinis* group (Figure 6C). Two OTUs (OTU 221 and OTU 236) assigned to the *S. pneumoniae* group were equally similar to all *Streptococcus* species in the *S. pneumoniae* group, with the exception of *S. pneumoniae* as five species in the *S. pneumoniae* group have identical sequences in the 16S rRNA V3–V4 region, whereas *S. pneumoniae* differs by one nucleotide. Two OTUs (OTU 203 and OTU 411) assigned to the *S. gordonii* group also showed a similar pattern: they were equally similar to three species in the *S. gordonii* group. As shown in this case study, many OTUs were indistinguishable among the species in the species group but were distinguishable among the species group. Most of the *Veillonella* OTUs were assigned to the *Veillonella parvula*, *Veillonella dispar*, or *Veillonella atypica* group (Figure 6D). In the *V. parvula* group, OTU 853 and OTU 276 were equally similar to multiple species in the group. These results might be inevitable when hypervariable regions are used at the species level. In addition, some novel species that are not stored in the 16S rRNA database but are equally similar to multiple known species could exist in the microbiome.

### A Case Study Using the Gut Microbiome of Colon Cancer Patients

**FIGURE 7:**
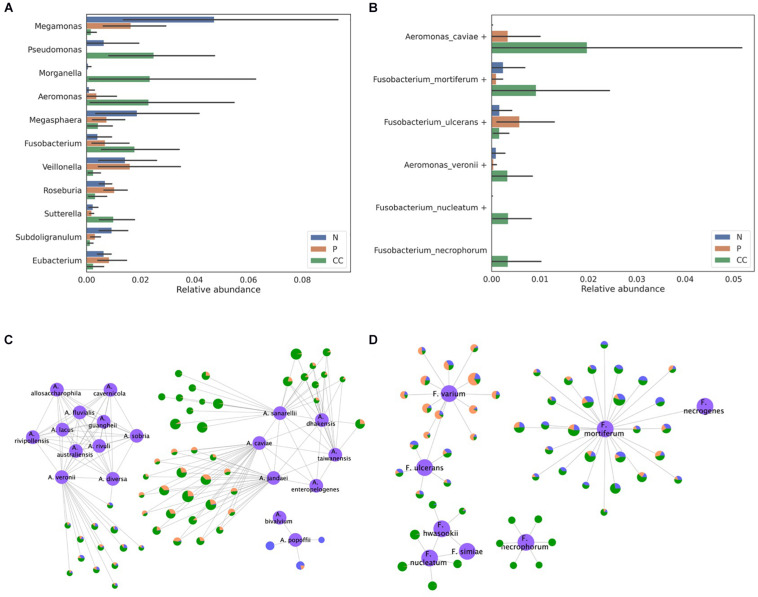
Classification of the bacterial composition of gut microbiome samples obtained from the stool of control, adenoma, and cancer patients. **(A)** The relative abundance of the genera that were found to have an abundance more than three times that of the average relative abundance in at least one pair among the control, adenoma, and cancer groups and **(B)** the relative abundance of the species belonging to the *Aeromonas* and *Fusobacterium* genera are presented. The assignment results for the **(C)** *Aeromonas* and **(D)** *Fusobacterium* genera are represented by the network. The reference sequences are colored in purple and labeled using their species name. Each node except the reference node represents one out, and the bacterial composition of samples from each type of patient is plotted as a pie-chart (blue for control, orange for adenoma, and green for cancer). All OTUs are represented as being proportional to their log-scaled size.

In total, 105 gut microbiome samples, consisting of 35 samples each from control, adenoma, and cancer patients, were downloaded from the SRA under the accession number SRP131074. *Bacteroides*, *Escherichia*, and *Prevotella* were reported as the most abundant genera in the previous study that analyzed these samples (Yang T. W. et al., 2019). Our results also showed that these three genera were the most abundant and in the same order (Supplementary Figure 7). Among the abundant genera, the abundance of *Megamonas*, *Pseudomonas*, *Morganella*, *Aeromonas*, *Megasphaera*, *Fusobacterium*, *Veillonella*, *Roseburia*, *Sutterella*, *Subdoligranulum*, and *Eubacterium* was found to differ by threefold between any two samples from the control, adenoma, and cancer groups (Figure 7A). Although most of the OTUs were assigned to a specific species without ambiguity, *Pseudomonas*, *Veillonella*, *Fusobacterium*, and *Aeromonas* OTUs were assigned to the *homologous species groups*. Notably, *Aeromonas* and *Fusobacterium* were the most abundant in the samples from the cancer group. For the *Aeromonas* OTUs, most of the dominant OTUs in the cancer group were assigned to either the *Aeromonas veronii* or *Aeromonas caviae* group (Figures 7B,C). Moreover, *Fusobacterium mortiferum*, *Fusobacterium necrophorum*, and *Fusobacterium nucleatum* were found to be abundant in samples from the cancer group, whereas *Fusobacterium ulcerans* was abundant in samples from the adenoma group (Figures 7B,D). Therefore, this indicates that our species-level profiling and network analysis based on *homologous species groups* could produce more specific and reliable information, which is higher resolution than the genus-level, to show differences in bacterial composition among patient groups.

## Conclusion

In the microbiome studies, one of the important tasks is profiling of the bacterial composition, which helps understand the biological functions of the microbiome. The species-level taxonomic assignment is critical, but an optimal solution has not been available thus far since the 16S rRNA sequences are highly homologous between the species in the same genus in many cases. We combined all the sequences from the GreenGenes, SILVA, and NCBI databases to include species that exist exclusively in each database. Even in the evaluation of genus-level taxonomy classification, the classifier trained with the sequences combined showed the best accuracy in terms of precision and recall rates. For each species, we constructed a consensus sequence model and determined *homologous species groups*, which was used for the species-level taxonomy assignment. The evaluation using simulated datasets and mock datasets showed a high correlation with the real bacterial composition. When analyzing real gut microbiomes, our method successfully performed species-level taxonomic assignment and identified differential abundance between different phenotypic groups.

## Data Availability Statement

The original contributions presented in the study are included in the article/Supplementary Material, re-annotated sequences and consensus sequences of each hypervariable region are available at https://sourceforge.net/projects/reannotated-16s-rrna-databases/files/.

## Author Contributions

H-JG designed and performed the data analysis and wrote the manuscript. MR designed and supervised the data analysis and wrote the manuscript. Both authors critically reviewed the manuscript and approved the final version.

## Conflict of Interest

The authors declare that the research was conducted in the absence of any commercial or financial relationships that could be construed as a potential conflict of interest.

## Footnotes

1 ftp://ftp.ncbi.nlm.nih.gov/genomes/all/GCF/

2 http://www.ebi.ac.uk/ena/data/view/PRJEB6244
