# Supplementary material for: Data-Driven Modeling for Species-Level Taxonomic Assignment From 16S rRNA: Application to Human Microbiomes
Source: Front Microbiol. 2020 Nov 12;11:570825. doi: 10.3389/fmicb.2020.570825 (PMC7688474; doi:10.3389/fmicb.2020.570825)
Supplement: Supplementary file 1 [file Data_Sheet_1.pdf]

Supplementary Material

1 Supplementary Figures and Tables

1.1 Supplementary Figures

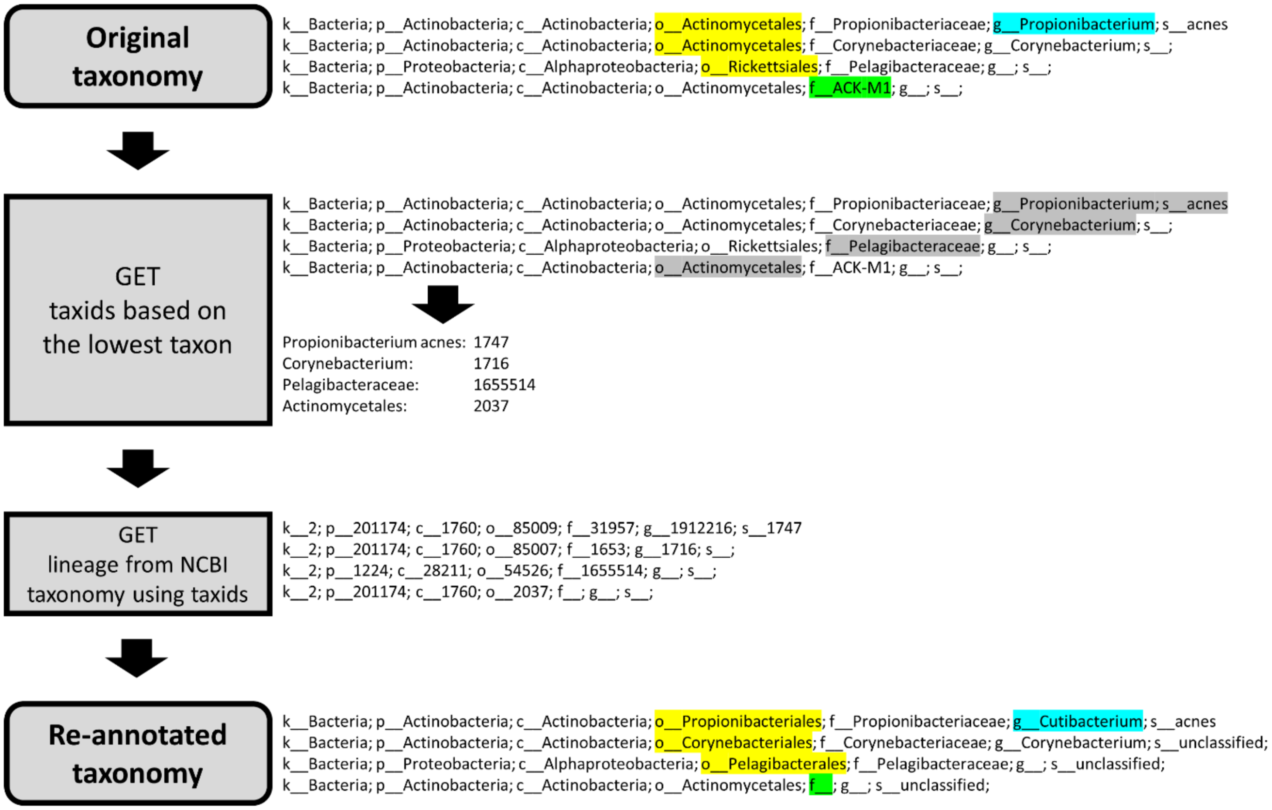

Supplementary Figure S1. A workflow and examples of re-annotation based on NCBI taxonomy classification. From the lineage provided in the original database, the lowest taxa present in NCBI lineage were shaded in gray and the re-annotated or removed taxa were shaded for each rank (yellow for order, green for family, cyan for genus). Each rank except for superkingdom was represented by their first letter ('k' for superkingdom, 'p' for phylum, 'c' for class, 'o' for order, 'f' for family, 'g' for genus, 's' for species).

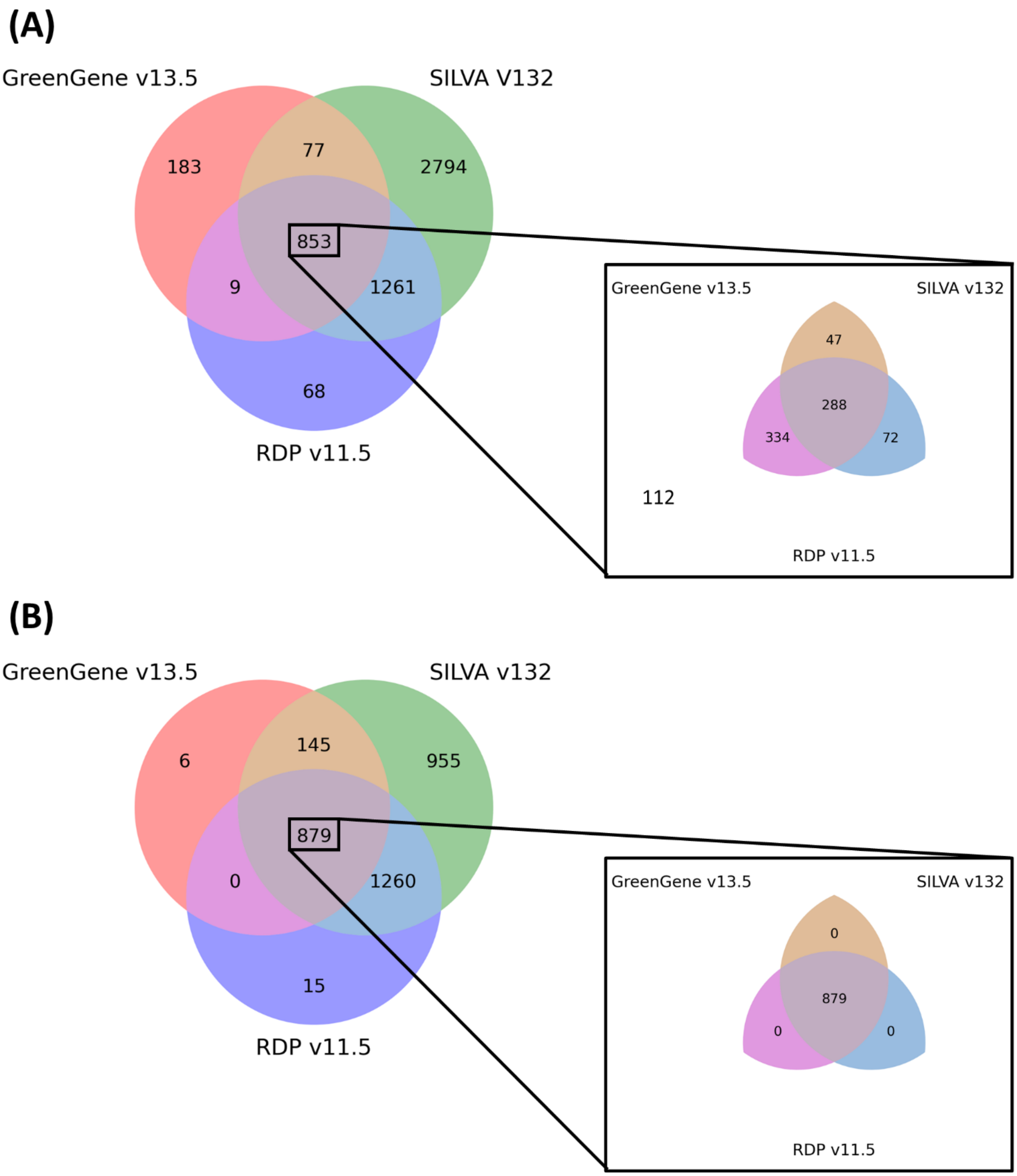

Supplementary Figure S2. The number of genera included in the three major 16S rRNA databases. (A) Before and (B) after the re-annotation of the lineage based on NCBI taxonomy. The subplot presents the number of genera whose lineage is annotated identically among three databases. Before re-annotating, lineages of only 288 genera were identically annotated and lineages of 112 genera were exclusively annotated among three databases.

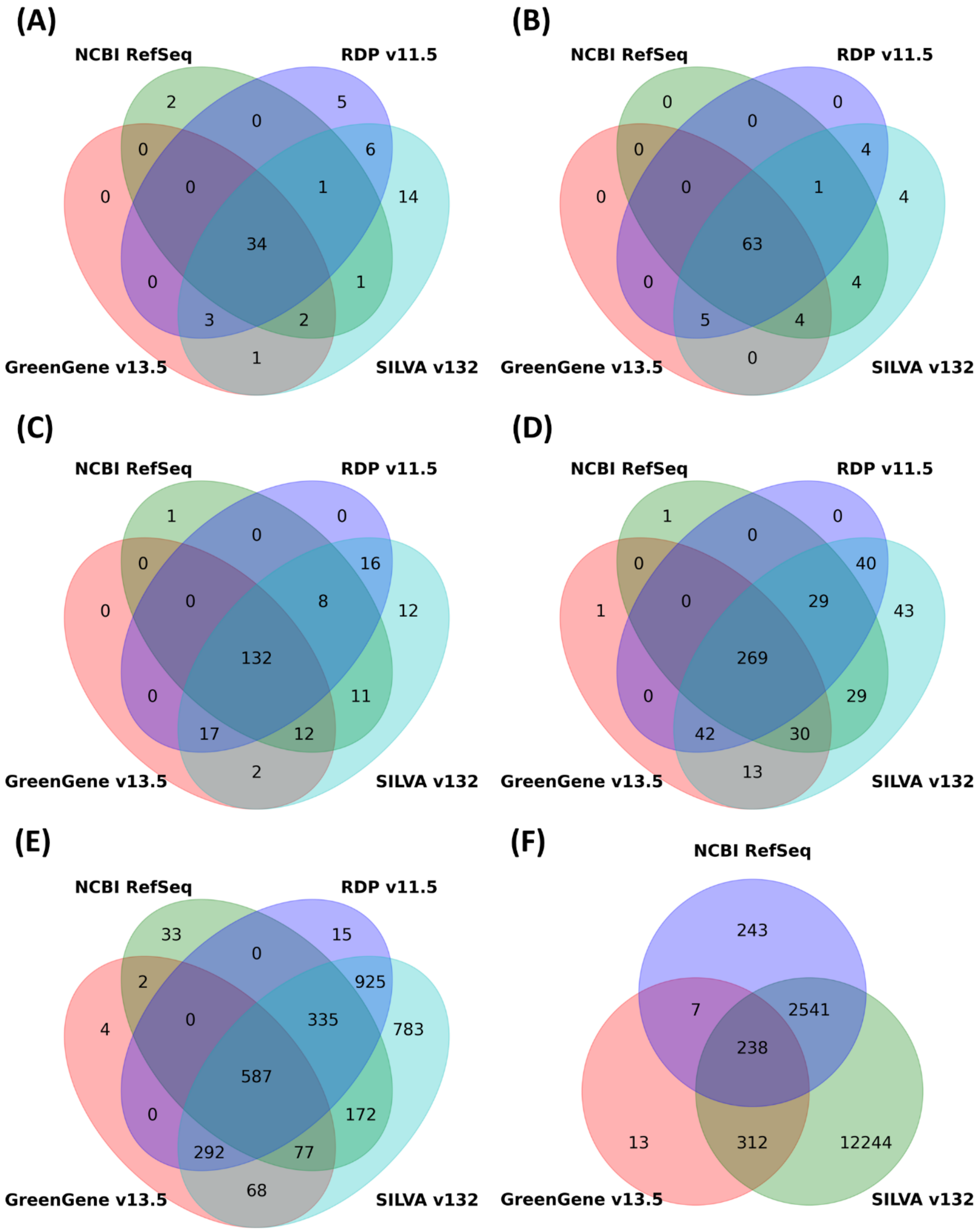

Supplementary Figure S3. The number of taxa included in the three major 16S rRNA databases and NCBI RefSeq database. The number of (A) phylum, (B) class, (C) order, (D) family, (E) genus, and (F) species were counted based on re-annotated taxonomy of three major 16S rRNA databases. The number of species was counted without the RDP database since the RDP database includes at most genus level labels.

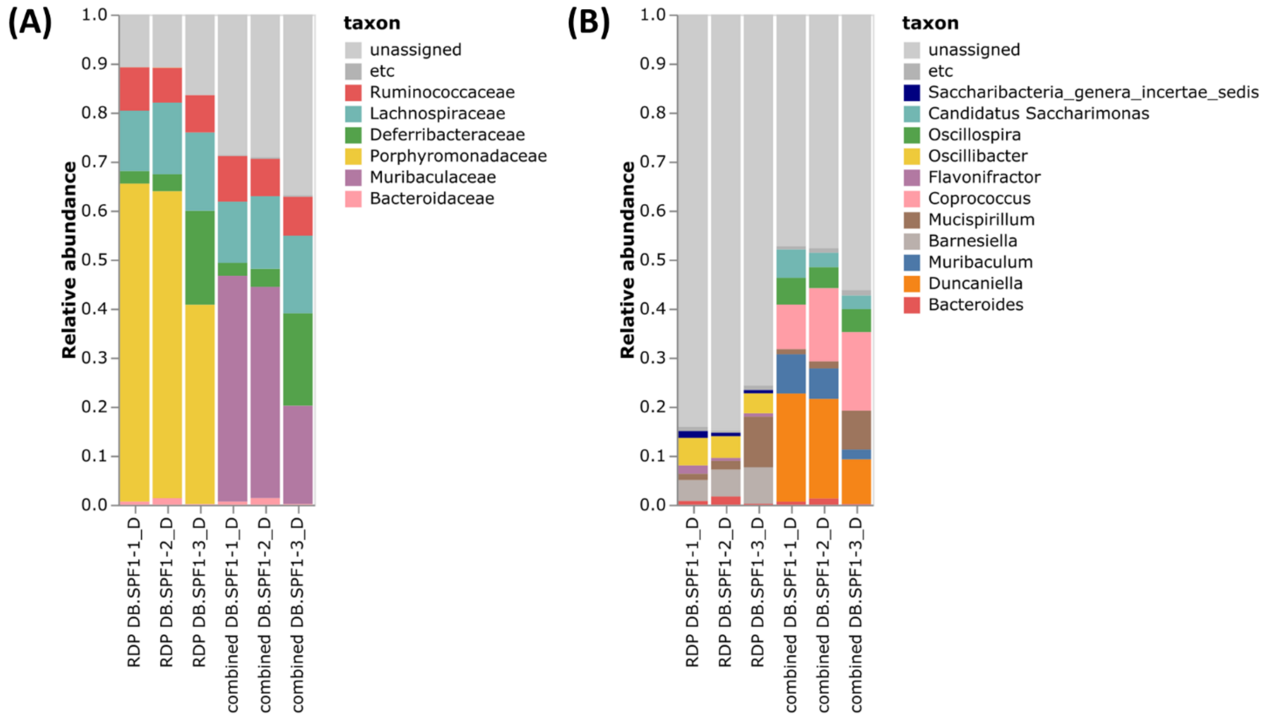

Supplementary Figure S4. A relative abundance of mouse gut microbiome at the (A) family and (B) genus level. To filter out misclassification, only bacteria that accounted for more than 1% in at least one sample were presented. The bacteria which were filtered out were aggregated to ‘etc’. *Duncaniella* (orange) and *Muribaculum* (navy) , which were misclassified as *Barnesiella* by the precompiled classifier, were successfully classified.

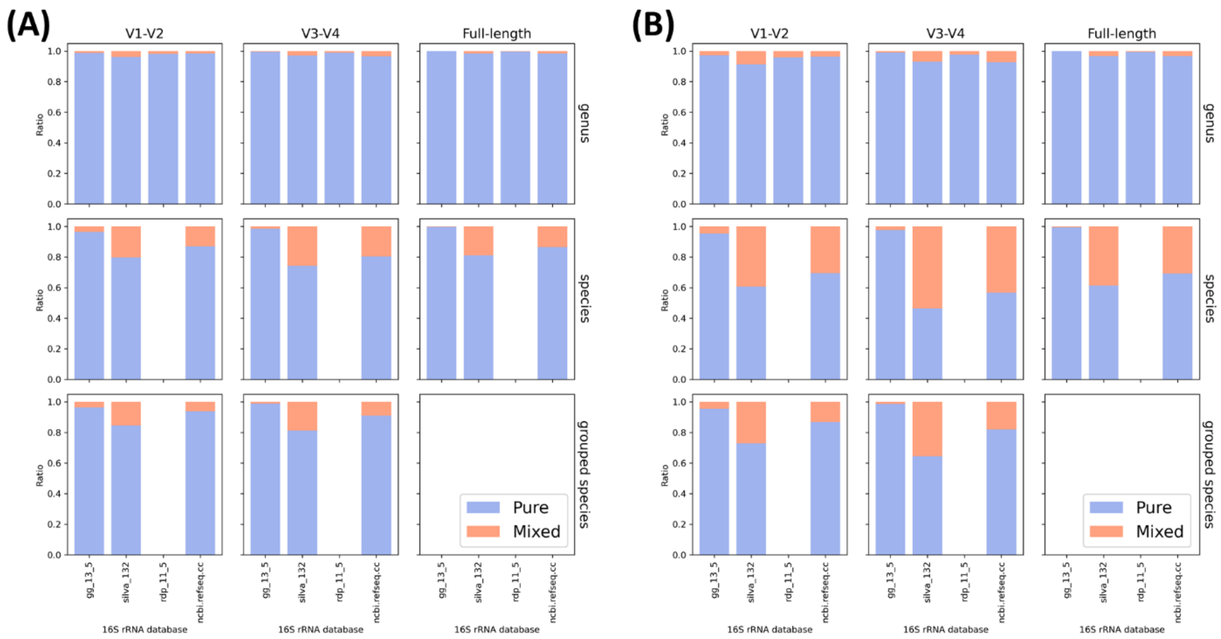

Supplementary Figure S5. A relative ratio of mixed (A) OTUs and (B) taxa. (A) OTUs containing multiple taxa were categorized as “Mixed”, otherwise “Pure”. Singleton OTUs were also categorized as “Pure”. (B) taxa clustered with different taxon sequences were categorized as “Mixed”, otherwise “Pure”.

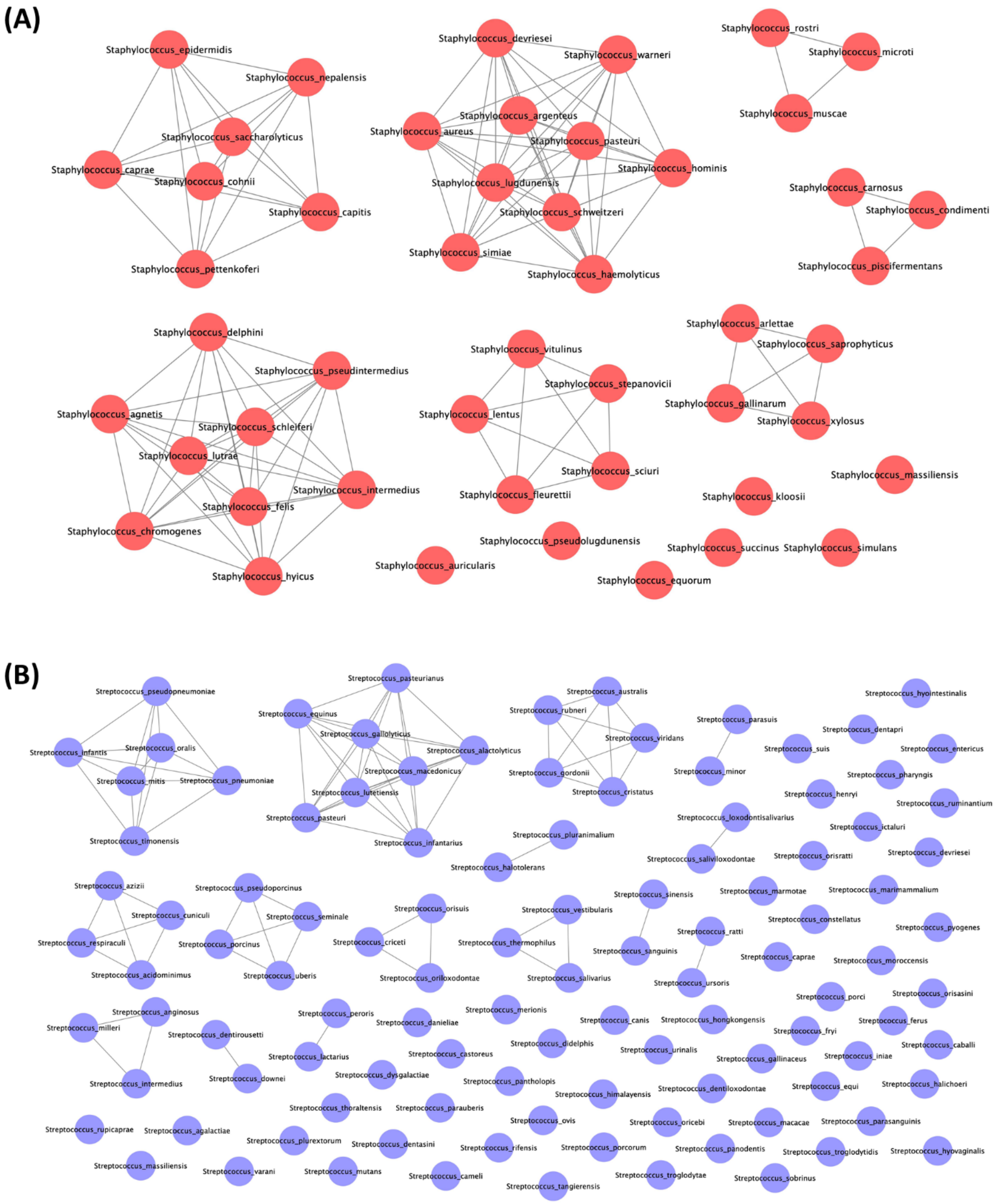

Supplementary Figure S6. Species networks of (A) *Staphylococcus* and (B) *Streptococcus*. Consensus sequences of each species were clustered with a 99% sequence similarity threshold. Each node represents each species and was colored according to the genus. The species which were clustered together were connected by an edge.

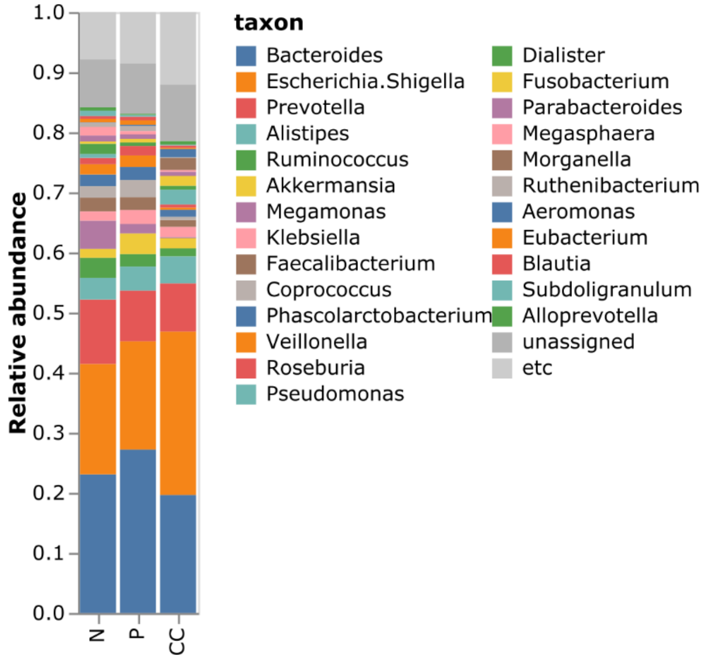

Supplementary Figure S7. An averaged relative abundance of top 25 genera for control (N), adenoma (P), and cancer (CC) samples. The unclassified samples at the genus level were labeled as 'unassigned' and all genera except top 25 genera were aggregated into the 'etc'.
